# Antimicrobial activity of bone cements embedded with organic nanoparticles

**DOI:** 10.2147/IJN.S86440

**Published:** 2015-10-12

**Authors:** Stefano Perni, Victorien Thenault, Pauline Abdo, Katrin Margulis, Shlomo Magdassi, Polina Prokopovich

**Affiliations:** 1 School of Pharmacy and Pharmaceutical Sciences, Cardiff University, Cardiff, UK; 2 Center for Biomedical Engineering, Massachusetts Institute of Technology, Cambridge, MA, USA; 3 Casali Institute, Institute of Chemistry, The Center for Nanoscience and Nanotechnology, The Hebrew University of Jerusalem, Jerusalem, Israel

**Keywords:** paraben, antimicrobial, bone cement, PMMA, brushite, hydroxyapatite

## Abstract

Infections after orthopedic surgery are a very unwelcome outcome; despite the widespread use of antibiotics, their incidence can be as high as 10%. This risk is likely to increase as antibiotics are gradually losing efficacy as a result of bacterial resistance; therefore, novel antimicrobial approaches are required. Parabens are a class of compounds whose antimicrobial activity is employed in many cosmetic and pharmaceutical products. We developed propylparaben nanoparticles that are hydrophilic, thus expanding the applicability of parabens to aqueous systems. In this paper we assess the possibility of employing paraben nanoparticles as antimicrobial compound in bone cements. The nanoparticles were embedded in various types of bone cement (poly(methyl methacrylate) [PMMA], hydroxyapatite, and brushite) and the antimicrobial activity was determined against common causes of postorthopedic surgery infections such as: *Staphylococcus aureus*, methicillin-resistant *S. aureus*, *Staphylococcus epidermidis*, and *Acinetobacter baumannii*. Nanoparticles at concentrations as low as 1% w/w in brushite bone cement were capable of preventing pathogens growth, 5% w/w was needed for hydroxyapatite bone cement, while 7% w/w was required for PMMA bone cement. No detrimental effect was determined by the addition of paraben nanoparticles on bone cement compression strength and cytocompatibility. Our results demonstrate that paraben nanoparticles can be encapsulated in bone cement, providing concentration-dependent antimicrobial activity; furthermore, lower concentrations are needed in calcium phosphate (brushite and hydroxyapatite) than in acrylic (PMMA) bone cements. These nanoparticles are effective against a wide spectrum of bacteria, including those already resistant to the antibiotics routinely employed in orthopedic applications, such as gentamicin.

## Introduction

Some microorganisms, a small fraction, can induce adverse effects on humans and are known as pathogens. The outcomes of an infection can range from pain and fever to death, depending on the microorganism and the physiological characteristics of the patient affected. Until the discovery of antibiotics by Alexander Fleming in the late 1920s, infections were almost untreatable and one of the most common causes of death; for instance, even a small superficial cut could have resulted in a fatality. Moreover, until that time surgical procedures had limited efficacy, as infections quickly developed in the majority of cases.

Antibiotics were hailed as the solution of infections; however, such beliefs were shattered by the evidence of microorganisms developing resistance against this treatment. It is now accepted that infectious microorganisms cannot be indefinitely treated with antibiotics.^1^,^2^ For many medical procedures to remain viable treatments, novel therapeutic approaches to infections need developing in order to guarantee sufficient protection against pathogens. The use of metals, predominantly silver, in the form of salts or nanoparticles is a well-established antimicrobial strategy that found applications in creams,^3^ wound wrappings,^4^ fabrics,^5^ and eluting surfaces.^6^–^8^ However, concerns regarding the use of silver are growing, particularly its long-term safety linked to accumulation in the environment and the body,^9^,^10^ hence other antimicrobial agents are in urgent need. Chitosan,^11^ honey extracts,^12^ and phytochemicals^13^ are natural compounds that have shown promising results. Paraben is another class of nonantibiotic antimicrobial compounds; paraben is the common name of an ester of *p*-hydroxybenzoic acid (Figure S1). They are a class of compounds with antibacterial and antifungal activity routinely used in cosmetics,^14^ pharmaceutical products,^15^ food products,^16^ and catheter lock solutions.^17^ The mechanism of action of parabens is thought to be the inhibition of the synthesis DNA and RNA or ATPases and phosphotransferases, and more recently, the impact on the capacity of the bacteria to withstand osmotic imbalance.^18^

Orthopedic surgeries are procedures that generally require anti-infection therapy; despite the risk posed by antibiotic resistance, these compounds are still the normal approach. Antibiotics can be delivered either parenterally^19^,^20^ or through elution from bone cement,^21^–^23^ when the latter is used, it provides a quick and strong attachment between bone fractures or bone and medical devices, like in the case of joint replacement procedures.^24^ Different types of bone cements are available and employed for different applications; for example, poly(methyl methacrylate) (PMMA) bone cement is used in joint replacements, providing high mechanical performance and a quick setting time.^24^ Calcium phosphate cements (CPC), in spite of the excellent osteoconductive properties, are used in low load-bearing conditions such as bone defect treatments, because of their low mechanical strength and brittle behavior.^24^ Hydroxyapatite and brushite are some of the allotropic forms of calcium phosphate and the two CPC that are currently used.^24^ Both types of bone cement are applied as a paste; however, their hardening processes are different: in the case of PMMA, polymerization takes place, while dissolution and precipitation occur for the setting of CPC.

Notwithstanding the antibiotic cover provided pre- and postorthopedic procedures, the incidence of infections is still relatively high, in some cases up to 9%–10%.^25^,^26^ The onset of infections not only causes pain and discomfort, but sometimes can even be life-threatening to patients; moreover, infections can considerably increase the cost of treatment due to a greater number of medications and lengthened hospital stays required.

In this paper, organic nanoparticles containing propylparaben were prepared through a recently described synthetic route, namely by solvent removal from a volatile oil-in-water microemulsion,^27^ and embedded in PMMA and calcium phosphate bone cement at different concentrations; their antimicrobial activity was determined against *Staphylococcus* spp. and *Acinetobacter baumannii* as model pathogens in postorthopedic infections. Once the effective concentration of these nanoparticles was determined, their effect on the mechanical and cytotoxic properties of bone cement was investigated.

## Materials and methods

### Chemicals and nanoparticles preparation

Propylparaben was supplied by Sharon Laboratories (Ashdod, Israel), while all other chemicals were purchased from Sigma-Aldrich (Gillingham, UK and Rehovot, Israel), unless otherwise stated, and solutions were prepared according to standard laboratory practice.

Organic nanoparticles were prepared according to the procedure developed by Margulis-Goshen et al.^27^ Briefly, a microemulsion composed of propylparaben 3% w/w, *n*-butyl acetate 3.5% w/w, *iso*-propyl alcohol 3.5% w/w, sodium dodecyl sulfate (SDS) 8% w/w, polyvinylpyrrolidone (PVP) 40,000 7% w/w, and water 75% w/w was spontaneously formed upon mixing of all components at room temperature. Fast, simultaneous removal of the solvents and water from the microemulsion by spray drying led to the formation of a fine powder composed of propylparaben nanoparticles, SDS, and PVP. The composition of the resulting powder was propylparaben 16% w/w, SDS 45% w/w, and PVP 39% w/w.^27^ The nanoparticles were readily dispersible in water, yielding a stable dispersion of particles with approximate diameter of 16 nm as indicated by small angle X-ray scattering, and had a zeta potential in NaCl 10 mmol of −46 mV.^27^ It was found that in this system, almost all propylparaben (about 98% w/w) was present as nanosized particles.^27^

### Bacteria

Gram-positive bacteria *Staphylococcus aureus* (NCIMB 9518), methicillin-resistant *S. aureus* – MRSA (NCTC 12493), and *Staphylococcus epidermidis* (RP62a) along with Gram-negative bacterium *Acinetobacter baumannii* (NCIMB 9214) were used. Bacteria frozen stokes were stored at −80°C; strains were streaked on BHI plates weekly (Oxoid Ltd, Basingstoke, UK) and incubated for 24 hours at 37°C, then stored at 4°C.

### Bone cement preparation and characterization

PMMA-based bone cement was obtained by mixing PMMA (Lucite International, Darwen, UK) (4.1 g), barium sulfate (0.46 g), benzoyl peroxide (0.1 g), methyl methacrylate (1.96 g), and *N,N*-dimethyl-*p*-toluidine (0.04 g).

Calcium phosphate-based bone cement was prepared according to the procedure described by Ewald et al.^28^ Hydroxyapatite bone cement was obtained by mixing sintered α-tricalcium phosphate (α-TCP) (Fluka, Gillingham, UK) (12 g) with Na_2_HPO_4_ 2.5% w/v (4 mL), while brushite bone cement was prepared by mixing sintered β-tricalcium phosphate (β-TCP) (6.62 g) with Ca(H_2_PO_4_)_2_ (5.38 g) and citric acid 0.05 M (4 mL).

For each bone cement, the solid and liquid phases were mixed in a beaker and poured into a mold that allowed the preparation of cylindrical specimens (6 mm in diameter and 12 mm in height), at an approximate setting time of 1 minute. The filled mold was pressed between two glass plates for 24 hours, and the cement was allowed to harden before the samples were extracted. Bone cement samples were stored in dark, sterile conditions (for no more than 3 days) prior to use.

The organic nanoparticles were added to the solid phase prior to mixing with the liquid phase, these were added with specific quantities to achieve a final concentration of 0.1%, 0.5%, 1%, 2%, 5%, and 7% w/w; bone cement of the appropriate type (PMMA, hydroxyapatite, or brushite) not containing nanoparticles was used as a control sample (0% w/w).

### Antimicrobial activity of organic nanoparticles and the bone cements

Approximately 15 mL of fresh sterile BHI broth (Oxoid Ltd) was inoculated with cells and incubated statically for 24 hours at 37°C.

The antimicrobial activity of the organic nanoparticles was compared with the activity of pure compounds (propylparaben, SDS, PVP) and their mixture before nanoparticles synthesis, determined through standard minimal inhibitory concentration (MIC) protocol.

Bone cement samples (cylindrical, 12 mm long, with a diameter of 6 mm) were placed in a 24-well plate, with a covering of 2 mL of the bacterial suspension (described before). The 24-well plate was incubated for 1 hour at 37°C statically, the bacterial suspension was removed and the samples were rinsed three times with fresh sterile phosphate buffer solution (PBS). Around 1 mL of a diluted solution of sterile BHI broth in PBS (1/10 BHI) was added to each sample, and the plate was incubated at 37°C. After 24 hours, 50 μL from each well was transferred to a 100-well plate (Bioscreen C; Labsystems Diagnostics Oy, Helsinki, Finland) containing 100 μL of fresh sterile BHI broth. The bacterial growth curves at 37°C were recorded every 15 minutes through optical density (OD) at 600 nm (OD_600_), using a plate reader (Bioscreen C analyzer; Labsystems Diagnostics Oy).

All tests were performed in triplicate and on three independent cultures, resulting in nine growth curves for each bacterium on each bone cement sample. Each growth curve was fit using the Gompertz growth model to extract values of lag phase and growth rate. Results are presented as mean and standard deviation.

### Water uptake

Bone cement samples containing paraben nanoparticles and controls were incubated in 5 mL PBS at 37°C for 3 months; for the first 2 weeks, the samples were weighed daily; after a fortnight the samples were weighed every 3 days.

### Compression testing of composite bone cements

Compression tests were performed according to BS ISO 5833:2002 on the Zwick Roell ProLine table-top Z050/Z100 materials testing machine (Zwick Roell, Ulm, Germany). Cylindrical samples 12 mm long with a diameter of 6 mm were employed. The compression tests were conducted at a constant crosshead speed of 20 mm/min to produce a curve of displacement against load. Tests were performed on bone cement samples with the following concentrations of organic nanoparticles (7% w/w for PMMA, 5% w/w for hydroxyapatite, and 1% w/w for brushite) freshly prepared and after 1 week immersion in PBS at 37°C. The compressive strength of the bone cement was determined by dividing the force applied to cause fracture by the original cross-sectional area of the cylinder. The average compressive strength of five specimens was calculated.

### In vitro cytotoxicity studies on composite bone cements

Osteoblast cells (MC-3T3) were cultured in Dulbecco’s Modified Eagle’s Medium (DMEM) supplemented with fetal bovine serum (10% v/v), cells were incubated at 37°C in humidified atmosphere with 5% CO_2_. Cells were grown until a 70% confluency was achieved, washed twice with sterile PBS, and detached with trypsin; osteoblast cells were counted (using trypan blue to differentiate between viable and nonviable cells) and diluted to a concentration 10^5^ cells/mL with fresh medium.

Prior to use, all bone cement samples (controls without nanoparticles and with organic nanoparticles 7% w/w for PMMA, 5% w/w for hydroxyapatite, and 1% w/w for brushite) were sterilized using 70% alcohol and washed three times with sterile PBS. Samples were placed in 24-well plates containing 2 mL of osteoblast cell suspension (prepared as described before). Osteoblasts were cultured on the bone cement samples at 37°C in humidified atmosphere with 5% CO_2_. The viability of osteoblast cells was assessed using the MTT enzyme assay protocol (Invitrogen, Paisley, UK). The MTT solution was prepared according to the manufacturer guidelines and 10 μL was added to each well. After incubation for 2 hours at 37°C in humidified atmosphere with 5% CO_2_, the samples were transferred to a sterile 24-well plate and the MTT solubilization solution was added. When full dissolution of the crystals occurred, 100 μL of liquid was transferred to a sterile 96-well plate, and the absorbance of each sample was read at 540 nm (OD_540_). Results are presented as the average and standard deviation of three independent bone cement samples.

### Propylparaben release from bone cement

Bone cement samples containing paraben nanoparticles, prepared as described in “Bone cement preparation and characterization” (with organic nanoparticles 7% w/w for PMMA, 5% w/w for hydroxyapatite, and 1% w/w for brushite), were incubated in 2 mL PBS at 37°C; the solution was replaced daily with fresh PBS and analyzed to quantify the amount of propylparaben released using reverse-phase HPLC. An Agilent series 1100 HPLC system was equipped with a Waters Spherisorb^®^ (Sigma-Aldrich, St Louis, MO, USA) 5 μm ODS2 (4.6×150 mm) analytical column thermostated at 25°C. The injection volume was 5 μL, the mobile phase was water:acetonitrile 50:50, with a flow rate of 1 mL/min, and the detector was a UV spectrophotometer at 254 nm. An example of a chromatogram for a 1 mg/mL solution of propylparaben in DMSO is shown in Figure S2; the calibration curve of the HPLC detection of propylparaben is presented in Figure S3.

### Bone cement settling time

The influence of the paraben nanoparticles on the bone cement settling time was determined through rheological tests using AR-G2 (TA Instruments, Hertfordshire, UK), using 40 mm Peltier plates. Dynamic oscillation tests were performed; in these measurements, a sinusoidal oscillation strain (σ) of small amplitude (σ_0_) and frequency (ω):

$$\sigma (t)=\sigma_{0}\;\exp(i\omega t)$$ (1)

was applied to the sample. The resulting stress (ω) was compared with the strain giving the complex modu lus G*.

$$G^{*}=\frac{\sigma (t)}{\gamma (t)}$$ (2)

Because the two sinusoidal waves will have a phase difference, δ, the storage (*G*′) and loss modulus (*G*″) can be defined as the component in phase and π/2 out of phase with the strain, respectively.

$$G^{*}=G^{\prime }+iG^{\prime \prime }$$ (3)

and

$$G^{\prime }=\left|G^{*}\right|\cos\;\delta$$ (4)

$$G^{\prime \prime }=\left|G^{*}\right|\mathrm{sen}\;\delta$$ (5)

Analysis was carried out using dynamic time sweep test that takes successive measurements at constant frequency and strain at selected intervals. The test was conducted at a strain of 0.1% and fixed frequency of 1 Hz.

All three types of bone cement containing 2% w/w of gentamicin were analyzed along with samples with paraben nanoparticles 7% w/w for PMMA, 5% w/w for hydroxyapatite, and 1% w/w for brushite. The two phases were mixed quickly with a spatula, the mixture was deposited onto the lower plate, and experiments started as fast as possible. To account for the time elapsed during mixing and pouring, the timer was started at the moment of mixing the liquid with powders.

Measurement of complex Young modulus and phase angle were taken every 6 seconds for up to 2,500 seconds. Each sweep experiment was carried out on three independently prepared cement samples, and results are presented as mean and standard deviation.

### Statistical analysis

The influence of paraben nanoparticles on mechanical and cytotoxic properties of bone cement was tested through ANOVA using SPSS (12.0) (SPSS Inc., Chicago, IL, USA). For all analyses, *P*<0.05 was considered statistically significant.

## Results

### Antimicrobial activity of bone cements containing nanoparticles

In general, it was found that the nanoparticles of propylparaben exhibited a significantly greater antimicrobial activity toward all bacteria tested than the propylparaben as raw material powder without conversion into nanoparticles. The nanoparticles were also found to be more potent than the physical mixture of all nanoparticle components, which has shown MICs about twice as high as the nanoparticles (Table 1). SDS and PVP alone did not exhibit antimicrobial activity at the concentrations corresponding to the quantities present at the MIC characteristic of the nanoparticles (data not shown).

**Figure 1:**
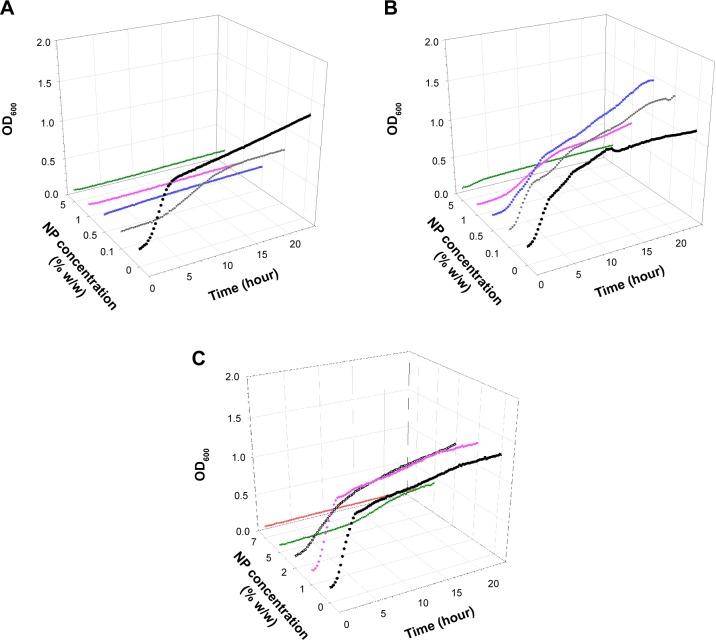
Examples of *Staphylococcus aureus* growth curves on (**A**) brushite, (**B**) hydroxyapatite, and (**C**) PMMA containing antimicrobial organic nanoparticles. **Notes:** • 0%, ▼ 0.1%, ■ 0.5%, ♦ 1%, ○ 2%, ▲ 5%, ▼ 7%. **Abbreviations:** NP, nanoparticle; OD_600_, optical density at 600 nm; PMMA, poly(methyl methacrylate).

**Figure 2:**
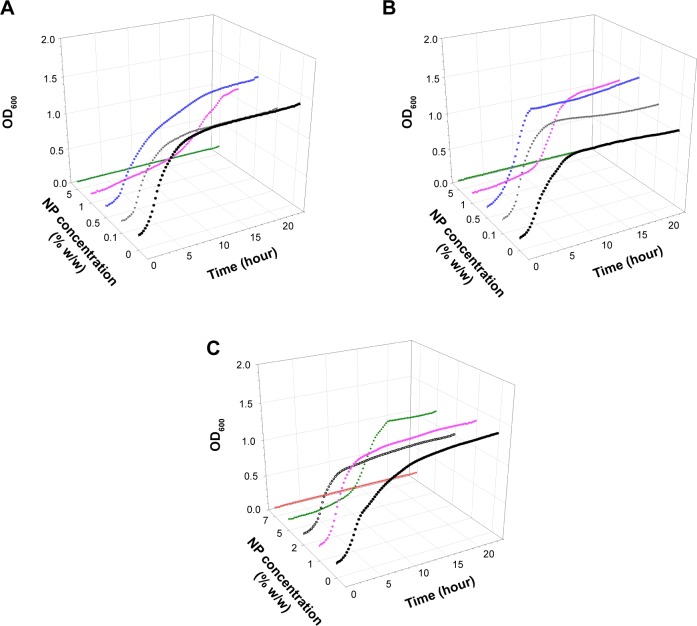
Examples of MRSA growth curves on (**A**) brushite, (**B**) hydroxyapatite, and (**C**) PMMA containing antimicrobial organic nanoparticles. **Notes:** • 0%, ▼ 0.1%, ■ 0.5%, ♦ 1%, ○ 2%, ▲ 5%, ▼ 7%. **Abbreviations:** MRSA, methicillin-resistant *Staphylococcus aureus*; NP, nanoparticle; OD_600_, optical density at 600 nm; PMMA, poly(methyl methacrylate).

**Figure 3:**
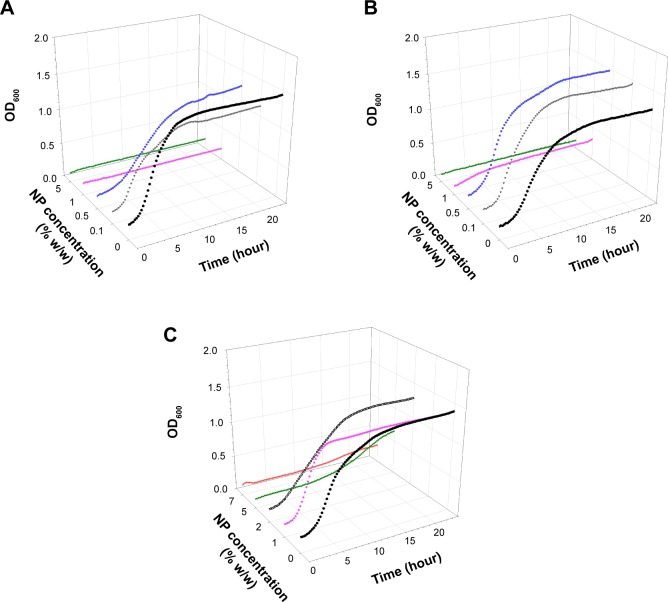
Examples of *Acinetobacter baumannii* growth curves on (**A**) brushite, (**B**) hydroxyapatite, and (**C**) PMMA containing antimicrobial organic nanoparticles. **Notes:** • 0%, ▼ 0.1%, ■ 0.5%, ♦ 1%, ○ 2%, ▲ 5%, ▼ 7%. **Abbreviations:** NP, nanoparticle; OD_600_, optical density at 600 nm; PMMA, poly(methyl methacrylate).

**Figure 4:**
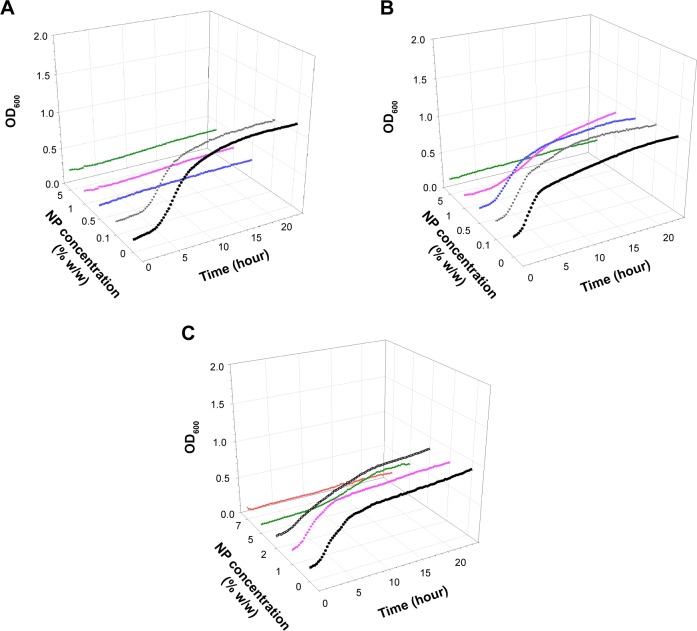
Examples of *Staphylococcus epidermidis* growth curves on (**A**) brushite, (**B**) hydroxyapatite, and (**C**) PMMA containing antimicrobial organic nanoparticles. **Notes:** • 0%, ▼ 0.1%, ■ 0.5%, ♦ 1%, ○ 2%, ▲ 5%, ▼ 7%. **Abbreviations:** NP, nanoparticle; OD_600_, optical density at 600 nm; PMMA, poly(methyl methacrylate).

Examples of growth curves for each of the bacteria tested on all bone cement samples are presented in Figures 1–4. In all cases, when no antimicrobial nanoparticles were present, the OD_600_ quickly started to increase (lag phase about 1–2 hours), reaching the stationary phase after 4–6 hours depending on the bacterium. With increasing concentration of nanoparticles, the lag phase duration expanded, when 1% w/w was added to brushite, no growth was detected for all bacteria but MRSA, hydroxyapatite containing 5% w/w achieved the same results, while 7% w/w of nanoparticles were required for PMMA bone cements.

Generally, 0.1% w/w of nanoparticles in brushite gave growth curves not dissimilar to control samples, apart from *S. aureus*; 0.5% w/w in hydroxyapatite and 1% w/w in PMMA were ineffective. The bacteria tested exhibited different responses to the antimicrobial compounds depending on the type of bone cement. For example, *A. baumannii* was the most affected by paraben in hydroxyapatite bone cement as it was the only one not able to grow with 1% w/w of nanoparticles, but was capable of surviving 0.5% w/w of nanoparticles in brushite bone cement, while *S. aureus* and *S. epidermidis* were not. MRSA was generally the least sensitive among the bacteria studied.

A more in-depth analysis of the growth curve is presented for each type of bone cement in Tables 2–4, where all growth rates are presented alongside the lag phases. It is evident that bacteria exposed to bone cements containing increasing concentrations of antimicrobial compounds exhibited generally slower growth rates due to a lack of growth detected.

### Cytotoxicity and mechanical properties of bone cements containing nanoparticles

**Figure 5:**
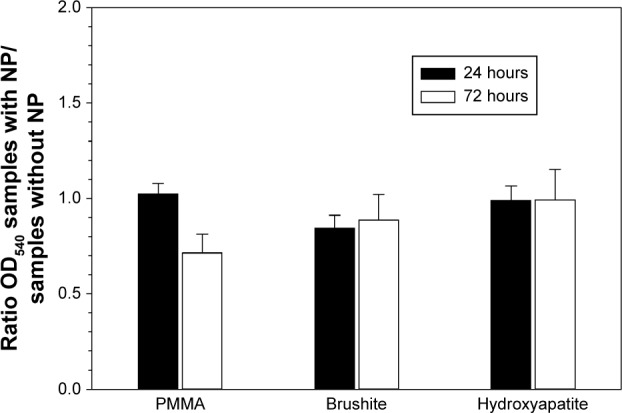
MTT assay for bone cements containing nanoparticles as ratio between OD_540_ of samples containing paraben nanoparticles (7% w/w for PMMA, 5% w/w for hydroxyapatite and 1% w/w for brushite) and control (same type of bone cement without nanoparticles). **Abbreviations:** NP, nanoparticle; OD_540_, optical density at 540 nm; PMMA, poly(methyl methacrylate).

**Figure 6:**
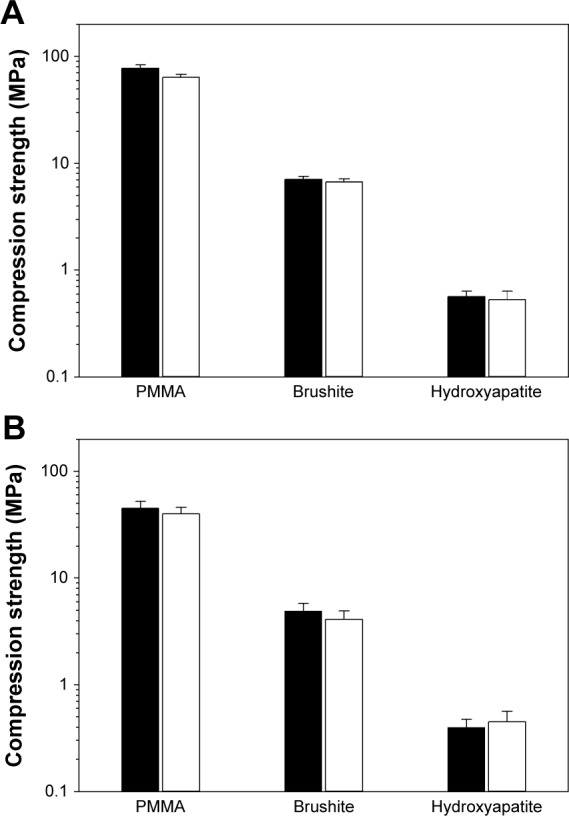
Compression strength of bone cements with 0% (control) and with organic nanoparticles (7% w/w for PMMA, 5% w/w for hydroxyapatite, and 1% w/w for brushite) freshly prepared (**A**), and after 7 days in PBS at 37°C (**B**). **Note:** Black columns represent control samples, and white columns represent bone cement containing nanoparticles. **Abbreviations:** PMMA, poly(methyl methacrylate); PBS, phosphate buffer solution.

The analysis of the possible influence of the paraben nanoparticles on the cytotoxic (Figure 5) and mechanical (Figure 6) properties of the bone cement revealed that concentrations capable of preventing infections (1% w/w for brushite, 5% w/w for hydroxyapatite, and 7% w/w for PMMA) had no adverse effects (*P*>0.05).

When immersed in fluids (PBS), the bone cement samples increased in weight during the first 4–5 days because of water uptake, and after that, the amount of fluid in the samples remained stable (data not shown). No difference was observed between the different concentrations of propylparaben nanoparticles encapsulated in bone cement (*P*>0.05). Furthermore, the water uptake results in a lower compression strength of all types of bone cement, regardless of the presence of propylparaben nanoparticles (Figure 6).

### Propylparaben release from bone cements

**Figure 7:**
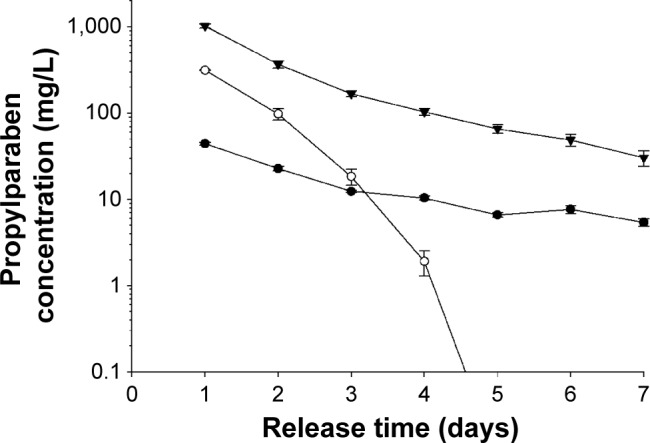
Concentration in the release medium of propylparaben from bone cements prepared with propylparaben nanoparticles. **Note:** • 7% w/w for PMMA, ○ 5% w/w for hydroxyapatite, and ▼ 1% w/w for brushite. **Abbreviation:** PMMA, poly(methyl methacrylate).

The release of propylparaben (Figure 7) from the samples containing the same amount of nanoparticles also indicated that the totality of the paraben is released from the calcium phosphate bone cement, but only about 5% of the initial amount of propylparaben is released from PMMA. Furthermore, the samples were releasing propylparaben continuously for the first 3–4 days. The amount of propylparaben released from hydroxyapatite and brushite was almost an order of magnitude higher than PMMA despite the initial concentrations in all three cases being very similar, and so was chosen as the minimum effective against the bacteria tested. Additionally, the concentration of propylparaben in the release medium for PMMA bone cement after 24 hours of elution, was closer to the MIC of the bacteria used in this study than the calcium phosphate bone cement that exhibited significantly higher concentrations of propylparaben than MIC.

### Settling times of bone cements containing nanoparticles

**Figure 8:**
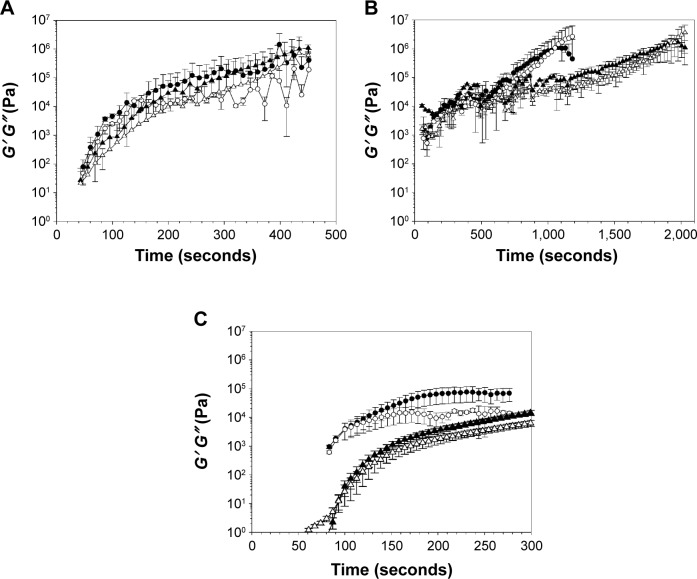
Storage (*G*′) and loss (*G*″) modulus of (**A**) brushite, (**B**) hydroxyapatite, and (**C**) PMMA bone cements containing 2% (w/w) of gentamicin (circles) or propylparaben nanoparticles 7% w/w for PMMA, 5% w/w for hydroxyapatite, and 1% w/w for brushite (triangles). **Note:** Full symbols (*G*′) and empty symbols (*G*″). **Abbreviation:** PMMA, poly(methyl methacrylate).

The possible influence of the organic nanoparticles on the kinetics of bone cement settling was investigated through the evolution of the rheological properties of bone cement dough after mixing (Figure 8). In all cases, the storage modulus (*G*′) was greater than the loss modulus (*G*″); the pattern followed a monotonic increase at an initial fast rate that slowed down reaching a plateau. For each type of bone cement, the presence of paraben nanoparticles required a slightly longer settling time (defined as the time needed for the dough to reach constant rheological properties). It was also evident that PMMA is the quickest type of bone cement to set (about 150 and 300 seconds for gentamicin and paraben containing bone cements, respectively), while hydroxyapatite required the longest (about 1,000 and 2,000 seconds for gentamicin and paraben containing bone cements, respectively).

## Discussion

### Parabens uses and safety

Propylparaben exhibits hydrophobic properties, therefore its use is limited to nonwatery systems; we have shown that the encapsulation of this drug in nanoparticles increases the hydrophilicity, resulting in stable dispersions and employability in aqueous environments thus expanding its possible applications. Despite the widespread applications of parabens, some concerns were raised regarding their potential safety as concentrations in environmental samples, human blood, breast milk, and tissues of these compounds had been steadily growing.^29^ Possible estrogenic effects have been suggested,^30^ and as they have been also found in breast cancer tissues, this led to the suggestion that parabens can adversely influence breast cancer formation.^31^,^32^ However, evidence of in vivo paraben-induced developmental and reproductive toxicity lacks consistency and physiological coherence as stated by Witorsch and Thomas.^33^ After many reviews and research, their use was found to be safe.^34^,^35^

### Infections and antimicrobial bone cements

The possibility of a microorganism to induce infection in a particular site of the body depends on its ability to survive and colonize that particular area; this is dictated by the environmental conditions of that location. The most common sources of postorthopedic infections are *S. aureus*, MRSA, and *S. epidermidis*;^36^,^37^ more recently, *A. baumannii* has given rise to concerns.^38^ The choice of bacteria tested in this work was based on such notions.

The antimicrobial protocols employed here are based on the assumption that bacteria attach to the bone cement sample during the initial contact with the suspension; cells capable of surviving the antimicrobial compound detach and are able to grow in the diluted broth.^7^,^8^,^39^ The growth curve using this suspension was recorded; the antimicrobial activity of the nanoparticles embedded in bone cement is positively linked to the length of the lag phase of the growth curves (Figures 1–4). Variations between samples are determined by the initial bacterial concentration in the broth containing the bone cement sample after 24 hours incubation post bacterial exposure. This is in virtue of the fact that cell concentrations below a certain threshold are not detectable through OD measurements, hence, the lower the initial cell concentration, the longer the time required to reach such cell numbers.^40^ Additionally, the decreasing growth rates of the surviving bacteria exposed to increasing concentrations of nanoparticles (Tables 2–4) demonstrated that the antimicrobial effect is not only limited to a reduction of the viable microbial population, but is also an indication that the viable cells did not exhibit the same phenotype of the cells in contact with the paraben nanoparticles. This slower growth rate could be attributed to irreversible cell damage or to the release of sublethal amounts of antimicrobial agents from the bone cement samples.

The efficacy of the paraben nanoparticles embedded in bone cement appeared to follow the pattern indicated below: brushite > hydroxyapatite > PMMA (Tables 2–4). This could be attributed to the different settling temperatures of the materials as this is one of the most significant differences between the two bone cement types. For CPC, this is generally the body temperature, while for PMMA the temperature can reach up to 70°C−80°C during settling. Polymerization is an exothermic reaction and is the leading cause for bone damage at the interface between bone cement and bone.^41^ Parabens are thermally stable^42^ and, therefore, temperature alone cannot be responsible for such decreased activity; however, radicals can interact with the paraben molecules and cause these molecules degradation, thus the active quantity remaining after bone cement settling is lower than the initial amount, requiring a greater quantity in PMMA bone cement to achieve the same results as in CPC. Furthermore, the different porosity of the bone cements can be a cause of the different antimicrobial agent release.

Gentamicin and tobramycin are the most common antibiotics used in PMMA bone cement^43^ in virtue of their thermal stability and broad spectrum; they are effective against β-lactam resistant strains such as MRSA. However, *S. epidermidis* strains such as RP62a and various *A. baumannii*, both tested in this work, are resistant to these drugs,^44^,^45^ rendering the use of these two antibiotics ineffective when such strains are involved. We tested the three types of bone cements supplemented with gentamicin 2% w/w (the commercial formulation of this antibiotic in bone cement) against the four pathogens, and the growth of *S. epidermidis* was not prevented (Figure S4). Our results demonstrated that organic nanoparticles made of propylparaben are effective against a wide range of bacteria, including antibiotic-resistant strains (Figures 3 and 4) found in orthopedic infections; hence, the use of these organic nanoparticles could offer not only a possible alternative to antibiotics, but also solve some of the problems already associated with antibiotic resistance. Moreover, the amount of paraben nanoparticles required to provide effective antimicrobial activity is similar to the amount of gentamicin and tobramycin used (2%–4% w/w) and is significantly lower than other antimicrobial agents, such as chitosan^11^ and quaternized chitosan derivative,^44^ that require about 20%–30% w/w.

Despite providing antimicrobial activity, in order to be a viable option, the organic nanoparticles must not induce negative effects on the other bone cement properties. For this reason, the cytotoxicity and compression strength of bone cements containing the amount of nanoparticles sufficient to exhibit antimicrobial capacity (7% w/w for PMMA, 5% w/w for hydroxyapatite, and 1% w/w for brushite) were determined. The results demonstrated that the nanoparticles did not have a detrimental effect on these two essential characteristics (Figures 5 and 6).

The release of propylparaben from the bone cement samples demonstrated a typical elution profile (Figure 7). However, the antimicrobial activity of the samples did not appear to be exclusively dependent on the amount of paraben released. For example, PMMA containing 7% w/w nanoparticles was as effective as 1% w/w in brushite, but returned a lower concentration in the medium. As the protocol to assess the antimicrobial activity employed in this work is based on the survival of the cells attached to the surface of the sample,^6^–^8^,^39^ then for a material to exhibit antimicrobial activity, it is not required for the drug in question to elute. The hydrophobicity of propylparaben is likely to be the reason for its low concentration in the medium when partitioning between PMMA and water. Additionally, no propylparaben remained embedded in the samples as the cumulative release reached 100% of the initial amount; however, this was not the case for PMMA where only about 5% was released. The entrapment of other antimicrobial compounds (such as antibiotics) in PMMA bone cement is a well-known phenomenon, and in our particular case, it could be a consequence of the hydrophobicity of propylparaben or of its inactivation during the polymerization.

### Material properties of bone cements

The time needed for the bone cement to develop the final mechanical properties (settling time) is a critical parameter that dictates operating procedures, both during application and after during patient recovery. Therefore, the introduction of the paraben nanoparticles into the bone cement formulation must not result in settling time greatly dissimilar from bone cement containing the commonly used gentamicin (2% w/w). We have proved (Figure 8) that the settling time of all three types of bone cements was slightly longer when paraben nanoparticles are present compared to gentamicin using rheological testing that is a standard procedure to investigate bone cement formulations. It appears that the use of the novel antimicrobial agents would not alter the already established procedures for the application of bone cement that are being employed. The profiles we detected are also comparable to those presented by others,^46^ particularly the similar values of *G*′ and *G*″ for PMMA bone cement.^47^

## Conclusion

Parabens are nonantibiotic antimicrobial compounds widely used in consumer products and considered safe as no satisfactory evidence has been found indicating any possible links to adverse effects.

We have demonstrated in this work that nanoparticles made from parabens can be used in bone cement to prevent the onset of infections. The efficacy depends on the type of bone cement, for example, calcium phosphate bone cements require a lower amount of parabens than the acrylic type (PMMA) in virtue of the lower settling temperature of the former. Our results prove that parabens could be employed in bone cement as alternatives to antibiotics, whose activity is gradually decreasing as a consequence of the rise in antibiotic-resistant microorganisms. Furthermore, the paraben nanoparticles are effective also against bacterial strains already resistant to some of the common antibiotics used in bone cements. No detrimental effect was detected on either compression strength or cytotoxicity of the bone cement when the paraben nanoparticles were added.

## Figures and Tables

**Table 1:** MIC (μg/mL) of pure propylparaben, organic nanoparticles, and hand mixture of nanoparticles components

Bacteria	NP	Mixture of NP components	Propylparaben
*S. aureus*	80	160	2,500
MRSA	80	160	1,250
*A. baumannii*	160	160	1,250
*S. epidermidis*	160	300	2,500

**Abbreviations:** MIC, minimal inhibitory concentration; MRSA, methicillin-resistant Staphylococcus aureus; NP, nanoparticles; S. aureus, Staphylococcus aureus; A. baumannii, Acinetobacter baumannii; S. epidermidis, Staphylococcus epidermidis.

**Table 2:** Lag phase and growth rate of the growth curves of survival on brushite bone cement containing organic nanoparticles

Concentration of nanoparticles (% w/w)	λ (hour)	Growth rate (hour^−1^)
*S. aureus*		
0	1.18±0.12	0.25±0.07
0.1	6.61±1.06	0.35±0.14
0.5	>24	No growth
1	>24	No growth
5	>24	No growth
MRSA		
0	0.91±0.28	0.27±0.06
0.1	1.32±0.17	0.28±0.03
0.5	2.27±0.35	0.31±0.04
1	10.00±1.11	0.09±0.01
5	>24	No growth
*A. baumannii*		
0	1.67±0.19	0.30±0.02
0.1	1.43±0.08	0.41±0.06
0.5	3.49±0.63	0.15±0.07
1	>24	No growth
5	>24	No growth
*S. epidermidis*		
0	3.29±0.71	0.18±0.03
0.1	4.13±0.31	0.19±0.03
0.5	>24	No growth
1	>24	No growth
5	>24	No growth

**Note:** Mean ± standard deviation.

**Abbreviations:** *S. aureus, Staphylococcus aureus; A. baumannii, Acinetobacter baumannii; S. epidermidis, Staphylococcus epidermidis*; MRSA, methicillin-resistant *Staphylococcus aureus*.

**Table 3:** Lag phase and growth rate of the growth curves of survival on hydroxyapatite bone cement containing organic nanoparticles

Concentration of nanoparticles (% w/w)	λ (hour)	Growth rate (hour^−1^)
*S. aureus*		
0	0.11±0.02	0.15±0.02
0.1	0.13±0.02	0.16±0.03
0.5	2.11±0.08	0.12±0.03
1	3.81±0.36	0.06±0.02
5	>24	No growth
MRSA		
0	1.25±0.22	0.27±0.03
0.1	1.44±0.26	0.31±0.05
0.5	2.25±0.35	0.31±0.04
1	10.02±0.73	0.26±0.07
5	>24	No growth
*A. baumannii*		
0	2.29±0.36	0.22±0.01
0.1	2.34±0.43	0.18±0.02
0.5	1.78±0.13	0.20±0.02
1	>24	No growth
5	>24	No growth
*S. epidermidis*		
0	1.60±1.24	0.17±0.00
0.1	1.23±0.55	0.14±0.01
0.5	2.12±0.25	0.14±0.02
1	8.45±1.27	2.42±5.22
5	>24	No growth

**Note:** Mean ± standard deviation.

**Abbreviations:** MRSA, methicillin-resistant *Staphylococcus aureus; S. aureus, Staphylococcus aureus; A. baumannii, Acinetobacter baumannii; S. epidermidis, Staphylococcus epidermidis.*

**Table 4:** Lag phase and growth rate of the growth curves of survival on PMMA bone cement containing organic nanoparticles

Concentration of nanoparticles (% w/w)	λ (hour)	Growth rate (hour^−1^)
*S. aureus*		
0	0.76±0.03	0.33±0.09
1	0.82±0.04	0.35±0.10
2	1.04±0.07	0.11±0.04
5	11.07±1.04	0.01±0.01
7	>24	No growth
MRSA		
0	0.82±0.16	0.18±0.01
1	1.23±0.11	0.27±0.06
2	1.19±0.13	0.24±0.04
5	9.39±0.67	0.28±0.07
7	>24	No growth
*A. baumannii*		
0	1.91±0.02	0.20±0.01
1	1.96±0.28	0.28±0.04
2	2.64±0.50	0.13±0.02
5	10.82±1.16	0.08±0.01
7	>24	No growth
*S. epidermidis*		
0	1.32±0.08	0.15±0.03
1	1.39±0.04	0.16±0.03
2	2.03±0.24	0.08±0.04
5	9.24±0.42	0.04±0.02
7	>24	No growth

**Note:** Mean ± standard deviation.

**Abbreviations:** MRSA, methicillin-resistant *Staphylococcus aureus*; PMMA, poly(methyl methacrylate); *S. aureus, Staphylococcus aureus; A. baumannii, Acinetobacter baumannii; S. epidermidis, Staphylococcus epidermidis*.
